# The role of maternal age and context-dependent maternal effects in the offspring provisioning of a long-lived marine teleost

**DOI:** 10.1098/rsos.170966

**Published:** 2018-01-10

**Authors:** Linsey M. Arnold, Wade D. Smith, Paul D. Spencer, Allison N. Evans, Scott A. Heppell, Selina S. Heppell

**Affiliations:** 1 Department of Fisheries and Wildlife, Oregon State University, Corvallis, OR, USA; 2 University of British Columbia, Vancouver, British Columbia, Canada; 3 Alaska Fisheries Science Center, NOAA, Seattle, WA, USA

**Keywords:** maternal effects, reproductive strategies, offspring provisioning, population dynamics, marine teleosts

## Abstract

Despite evidence of maternal age effects in a number of teleost species, there have been challenges to the assertion that maternal age intrinsically influences offspring quality. From an evolutionary perspective, maternal age effects result in young females paradoxically investing in less fit offspring despite a greater potential fitness benefit that might be gained by allocating this energy to individual somatic growth. Although a narrow range of conditions could lead to a maternal fitness benefit via the production of lower quality offspring, evolutionary theorists suggest these conditions are seldom met and that the reported maternal age effects are more likely products of the environmental context. Our goal was to determine if maternal effects operated on offspring provisioning in a long-lived rockfish (genus *Sebastes*), and to evaluate any such effects as an intrinsic function of maternal age or a context-dependent effect of the offspring release environment. We found that offspring provisioning is a function of both maternal age and the timing of offspring release; older females exhibit increased provisioning over younger females throughout the spawning season despite a decrease in provisioning across all maternal ages as the season progresses. These findings suggest a role for both maternal age effects and a potential context-dependent maternal effect in population productivity, carrying important implications when modelling population persistence and resilience.

## 1. Introduction

Maternal effects are a driving determinant of offspring phenotype documented in a large array of taxa spanning the plant and animal kingdoms [1]. Defined as the causal influence of the maternal phenotype on the offspring phenotype [2], maternal effects are suggested to be more influential than genotype or the extra-maternal environment [3]. Offspring provisioning, brood protection, oviposition site, dispersal and sex determination are a few of the well-documented examples of maternal effects, but recent evidence suggests a role for maternal age in the expression of maternal effects [4]. In a senescence framework, the transgenerational fitness effects of maternal age are negative [5,6]; however, in marine teleosts exhibiting indeterminate growth and ‘negligible senescence’ [7], the positive effects of increased maternal age are documented in a minimum of 29 fish species representing the broad taxonomic diversity of 15 families [8]. Despite the increasing research devoted to the positive impact of female age on offspring quality in marine teleosts, the nature of the maternal age effect remains unclear. Whether there exists an intrinsic link between maternal age and offspring quality or whether the observed maternal age effect results from an interaction between maternal age and the offspring environment is unknown [9].

From an evolutionary perspective, maternal effects are reproductive strategies that enhance offspring survival; hence, they increase maternal fitness. The classic model of offspring size evolution from Smith & Fretwell [10] optimizes the parental investment per offspring to maximize parental reproductive success, with the optimal offspring size–number trade-off informed by the offspring size–fitness relationship [11]. This simple model assumes a constant environment and does not consider how maternal age affects the offspring size–fitness relationship. Using a dynamic state-variable model, Kindsvater *et al*. [12] showed that when survival costs of reproduction depend on total reproductive effort, young females minimize expense by reducing offspring size instead of number, whereas older females increase offspring size. However, a model developed to investigate maternal age effects in marine fishes by Marshall *et al*. [9] showed a narrow range of conditions leading to a maternal fitness benefit via production of lower quality offspring. This finding builds upon the models of Parker & Begon [13] that suggested no net fitness benefit of an increase in offspring size between small and large females due to an increasingly competitive offspring environment with increasing clutch size in terrestrial insect species. However, an empirical test of the models hypothesized by Parker & Begon [13] found little support for sibling competition driving the relationship between maternal and offspring size in an arborescent bryozoan [14]. Indeed, sibling competition effects have not been readily apparent in marine fish species with pelagic eggs and larvae, which are generally considered to experience density-independent mortality [15]. Marshall *et al*. [9] contend that maternal age effects result in young females paradoxically investing in less fit offspring, despite the greater fitness benefit that might be gained from allocating this reproductive effort to somatic growth. Therefore, the more likely explanation for the observed positive effect of maternal age on offspring fitness in marine fishes is probably a context-dependent relationship between maternal age, environment and offspring provisioning.

In the genus of live-bearing rockfishes (*Sebastes*) of the northeast Pacific, the offspring of older females are provisioned with larger oil globules, improving survival in laboratory settings through an increased time to starvation and increased growth rates [16–18]. However, the results of controlled laboratory experiments could be misleading if the fitness benefit of the maternal effect is dependent upon the environmental context, a context-dependent maternal effect [19]. Indeed, studies of rockfish reproductive behaviour conducted in field settings show older females spawn (hereafter ‘parturiate’) earlier or over a more protracted season [17,20–22]. In the context of a variable oceanic environment, releasing offspring at different times could equate to offspring released in different environments, suggesting the maternal age effect evident in controlled experimental settings may instead be an environmentally driven, context-dependent maternal effect when observed in a field setting. In a field study investigating both offspring provisioning and parturition timing, Fisher *et al*. [23] found increased offspring provisioning among species of rockfish that release offspring in winter versus late spring. The authors hypothesized this difference could be attributed to seasonal changes in the California Current, which is typified by low productivity in the winter, favouring increased larval provisioning, and high productivity in the late spring, favouring decreased larval provisioning.

Because older females can inherently produce more offspring due to indeterminate growth [8,24], age-specific maternal effects imply greater fitness for older females based on their potential for disproportionate contributions to the next generation. Additionally, context-dependent maternal effects, in which offspring provisioning varies in response to environmental condition, may stabilize a population's reproductive capacity (i.e. the production of viable offspring [25,26]) if old and young females release larvae at different times and environmental conditions. Estimating and predicting population productivity is key to the long-term viability of fisheries resources [27], particularly for a long-lived species with highly episodic recruitment of young to the population. Our goal was to determine if the maternal age effects noted in an increasing number of teleosts were also present in a long-lived rockfish of both ecological and commercial significance, and to further evaluate the nature of the maternal effects as intrinsic effects of maternal age or context-dependent effects of release environment.

## 2. Material and methods

### 2.1. Sample collection

**Figure 1.**
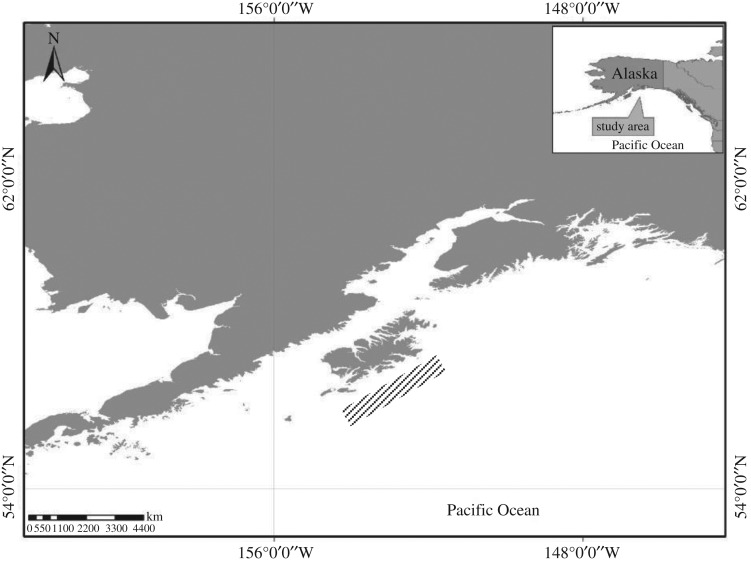
Pacific ocean perch (*Sebastes alutus*) sampling locations in the northern Gulf of Alaska, south of Kodiak Island (diagonal lines). Sampling locations intentionally vague.

For this work we selected the Pacific ocean perch (POP, *Sebastes alutus*) in the Gulf of Alaska. POP are a deep-dwelling commercially harvested species with a lifespan of 100 years [28]. As with all rockfish, POP are live-bearers and release developed larvae in a single batch during the spring parturition season beginning in April and proceeding through May. In 2006–2008, we sampled POP across a six-week period during the parturition season from the slope waters of the Gulf of Alaska south of Kodiak Island aboard the chartered fishing vessel *Gold Rush* (figure 1). In 2006, trawl tows were conducted in Julian weeks 18, 20 and 22. In 2007, trawl tows were conducted in Julian weeks 16, 17, 19 and 21. Sampling in 2008 was conducted in Julian week 20. Locations and depths of all trawl tows were based on the knowledge of the captain, with sampling focused in areas for which the captain's fishing records indicated high concentrations of POP during the parturition period. After each tow, female POP were measured (total length, nearest cm) and assigned a reproductive status (1. immature, 2. maturing and vitellogenesis, 3. fertilized eggs and eyed larvae, 4. post-parturition) following visual examination of the gonads. Otoliths and gonads were collected from all individuals. Larvae and gonads were preserved at sea in 10% buffered formalin.

### 2.2. Age determination

Ages were estimated from band counts of otoliths using the break-and-burn method [29]. All ageing was done by scientists at the National Oceanic and Atmospheric Administration (NOAA), National Marine Fisheries Service (NMFS), Alaska Fisheries Science Center in Seattle, Washington. All females collected in 2006 (*n* = 412) were aged. A subsample of females were aged for 2007 (*n* = 174) and 2008 (*n* = 140). Samples were binned into 1 cm size classes and a maximum of 10 adult fish in each size class were aged.

### 2.3. Gonad histology

To confirm the at-sea visual examination of gonad development, histological sections of gonads were prepared using standard techniques [30]. Each slide was viewed under light microscopy at 4× to 40× magnification, and developmental stage was determined using criteria adapted from Wyllie-Echeverria [31], Bowers [32] and Chilton [33].

### 2.4. Larval development and oil globule volume

**Figure 2.**
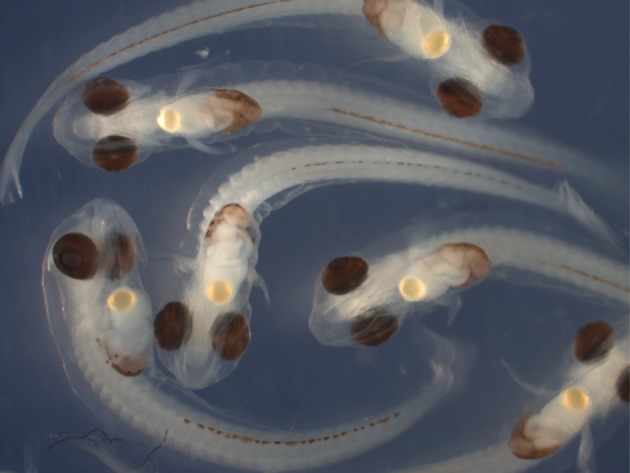
Pre-parturition larvae (stage 32) defined by hatching, gut formation, and much reduced oil globule volume.

Larval oil globule volume (OGV) was considered a proxy for larval quality, as OGV was correlated with the time to 50% mortality of larvae in a similar rockfish species [18]. Larvae were photographed with a Leica SP8 APO integral dissecting camera scope, and the OGV was measured using image analysis software ImageJ [34]. Because larvae consume the oil globule throughout the developmental period, OGV for each female's larvae was determined only for larvae in the latest stage of development, stage 32, following criteria developed by Yamada & Kusakari [35] (figure 2) to standardize comparisons among individuals. Up to 20 larvae per female were measured and OGV measurements were obtained from a total of 1084 larvae collected from 58 females in years 2006 and 2007. Due to compromised sample storage, larvae collected in 2008 were not included in the OGV analysis.

### 2.5. Data analysis

To evaluate parturition timing with respect to maternal age, the proportion of fish in each of the four reproductive stages defined above was calculated for ‘early’ and ‘late’ parturition periods binned by ‘young’ and ‘old’ age categories. We defined the early parturition season by Julian weeks 16, 17 and 18; and the late parturition season by weeks 20, 21 and 22. The maternal age defining young versus old females, less than or equal to age 14, was set to the estimated age at which 95% of POP females are mature, following the maturity ogive in the most recent Gulf of Alaska POP stock assessment [36].

Previous research on rockfish parturition timing has found older females begin releasing larvae earlier in the season than younger females [17,21,22]. As such, we modelled larval OGV as a function of both maternal age and parturition timing. Graphical analysis of the standardized residuals obtained by a fixed effects linear regression model revealed a female effect on OGV (i.e. the OGV varied non-randomly between females after adjusting for age and parturition timing). Therefore, oil globule volume was modelled with a linear mixed effects model that controlled for female of the form:

2.1 $$\text{OG}{\text{V}}_{ij}=\alpha +\beta_{1}X\ldots \beta_{6}X+f_{j}+\varepsilon_{ij}\;\text{where}\;f_{j}∼N(0,\sigma_{f}^{2})\text{ and }\varepsilon_{ij}∼N(0,\sigma_{R}^{2}),$$

where OGV*_ij_* was the oil globule volume of larvae *i* from female *j*, *α* was a fixed effect intercept term and *β_n_* were the regression coefficients of the *X* fixed effect variables that included maternal age, parturition week, year and all interactions. Age and week were treated as numerical values and were centred with respect to their means prior to modelling to avoid multicollinearity between these variables and their interactions. The offset intercept term, *f_j_*, varies between females and was modelled as a normally distributed random effect with a mean of zero and variance of $\sigma_{f}^{2}$, whereas the random deviates, *ϵ_ij_*, were assumed to be normally distributed with mean zero and variance $\sigma_{R}^{2}$. The biological context of a female random effect concerns factors unique to individual females, such as physical condition, that could result in the OGV of larvae from the same female being more similar to each other than to those of larvae from other females. Both inter- and intra-annual variability is captured by including both Julian week and year in the model as fixed effects.

Following an information-theoretic approach for model selection [37], we evaluated two candidate models relating maternal age and parturition week to larval quality. The candidate set included a linear regression model with no female random effect and a linear mixed effect model with a varying intercept by female (equation (2.1)). We fit the models using a maximum-likelihood approach and compared Akaike's Information Criteria [38] with a bias adjustment for small sample size, AICc [39]. Akaike weights determined the model with most support relative to the candidate model set. Model validation included assessment of residual homogeneity, independence and normality following the methods of Zuur *et al*. [40]. Additionally, we calculated marginal and conditional *R*^2^ values following Nakagawa & Schielzeth [41] to assess the variance explained by including a female random effect.

## 3. Results

A total of 1355 POP were collected during our 3-year investigation. Ages of females ranged from 4 to 32 years. Fifty-eight females from 2006 and 2007 had ovaries with pre-parturition larvae (larval developmental stage 32, figure 2) for calculation of oil globule volume.

### 3.1. Parturition timing and development

**Figure 3.**
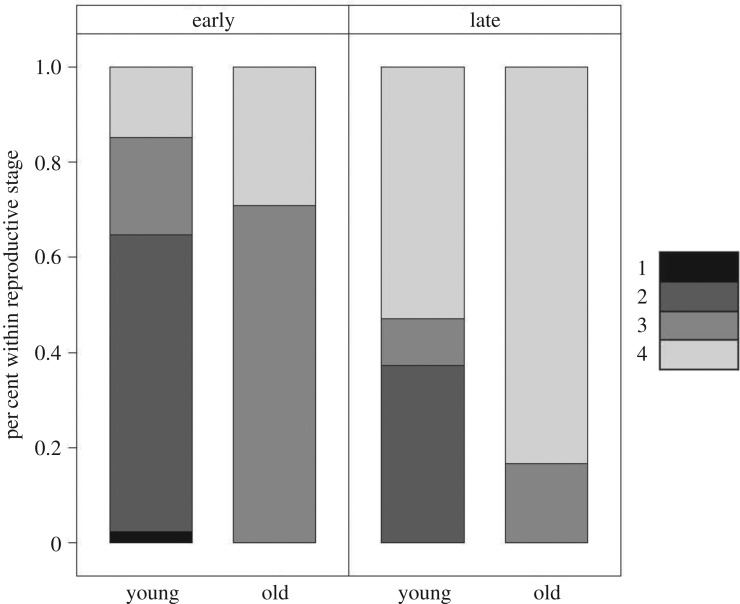
Reproductive stage of old (15+ years) and young females early (weeks 17–19) and late (weeks 20–22) in the sampling period. Stage 1 is immature, stage 2 is maturing and vitellogenesis, stage 3 is fertilized eggs and eyed larvae, stage 4 is post-parturition. In the early and late categories, there were 88 and 191 young females, respectively, and 24 old females included in each of the timing categories.

Early in the parturition season (late April to mid-May), 25% of young POP contained eyed larvae, the most advanced stage of development prior to parturition. However, during the same time period, 70% of age 15+ POP had eyed larvae with the remaining 30% at the post-parturition stage (figure 3). Post-parturition ovaries were observed first in the oldest fish, and accounted for 83% of the old female samples late in the parturition season, whereas only 55% of young females were at the post-parturition stage. The earliest stages of oocyte development were observed in only the youngest fish throughout the sampling period.

Asynchronous development between the two lobes of the ovary was found in 3.2% of the observed specimens for which reproductive status was assessed from both ovaries. When encountered, this variation between ovaries was evident both macro- and microscopically. These differences were most evident among specimens which possessed one apparently spent, resting, or non-functional ovary while the other contained eyed larvae. A single hermaphroditic specimen was also observed.

### 3.2. Larval quality

**Figure 4.**
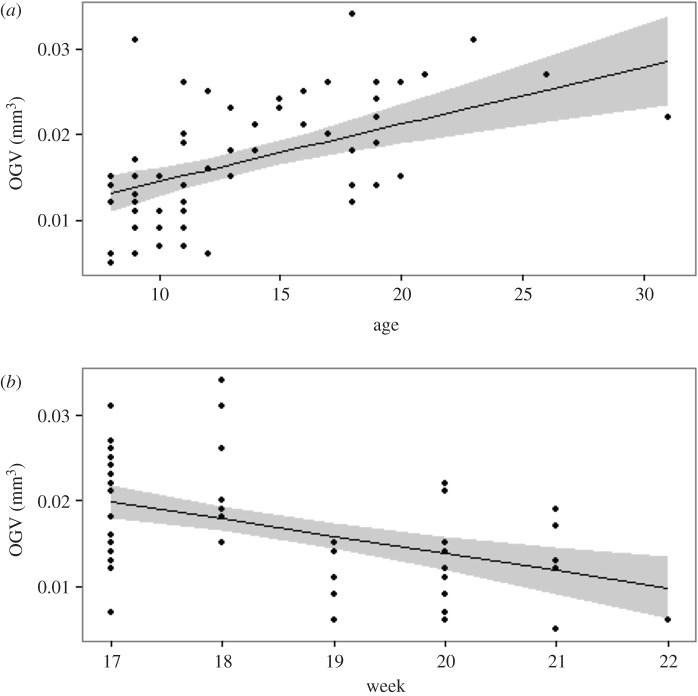
Relationships between oil globule volume (OGV), maternal age (*a*), and week of parturition (*b*). Grey bands represent the 95% confidence interval.

Older females provisioned their larvae with larger oil globules than younger mothers (figure 4*a*) and larvae released earlier in the parturition season were provisioned with larger oil globule volumes than larvae released later in the season (figure 4*b*). Of the two candidate models, the linear mixed effects model best described the relationship between OGV, maternal age, parturition week and year:

3.1 $$\begin{array}{l} \text{OG}{\text{V}}_{ij}=0.02+0.0007\text{Ag}{\text{e}}_{j}-0.004\text{Wee}{\text{k}}_{j}-0.004\text{Yea}{\text{r}}_{j}+0.003\text{Wee}{\text{k}}_{j}\text{Yea}{\text{r}}_{j}+f_{j}+\varepsilon_{ijk}\\ \text{where}\;\;\varepsilon_{ijk}∼N(0,\;\sigma_{R}^{2}*s_{k}^{2})\;\;\text{and}\;\;f_{j}∼N(0,\sigma_{f}^{2}) \end{array}$$

where $s_{k}^{2}$ allowed for different within-female variance for each week, *k*, in order to resolve heterogeneity in the residuals. The delta AIC value between the two candidate models (669 units) supports the inclusion of a female random effect (table 1). The model showed a significant effect of maternal age, parturition week, year, and the interaction of parturition week and sampling year on larval OGV (table 2). The marginal and conditional *R*^2^ values were 0.61 and 0.78, respectively, meaning the covariates of maternal age, parturition timing, and year explained 61% of the variation and the fixed effects plus the female random effect explained 78% of the variation in larval OGV.

**Table 1.** Akaike information criterion (AIC) from two candidate models applied to Gulf of Alaska Pacific ocean perch larvae (*n* = 1084). The log-likelihood (log*L*), AICc and ΔAICc measures determine the Akaike weights (*w*) which indicate the model with the greatest relative support.

candidate model	fixed effects	random effect	log*L*	AICc	ΔAIC_c_	*w*
linear mixed effects	age, week, year, week*year	female	4177	−8329	0	1
linear regression	age, week, year, week*year		3836	−7660	669	0

**Table 2.** Summary statistics of the fixed effect coefficients for the linear mixed effects model describing the relationship between larval oil globule volume (OGV), maternal age and parturition week with a random female effect.

covariate	value	s.e.	d.f.	*t*	*p*
(intercept)	0.0205	0.0013	1026	15.9	<0.001
age	0.0007	0.0001	53	5.7	<0.001
week	−0.0044	0.0009	53	−5.0	<0.001
year	−0.0042	0.0015	53	−2.8	0.0069
week*year	0.0028	0.0010	53	2.7	0.0089

**Figure 5.**
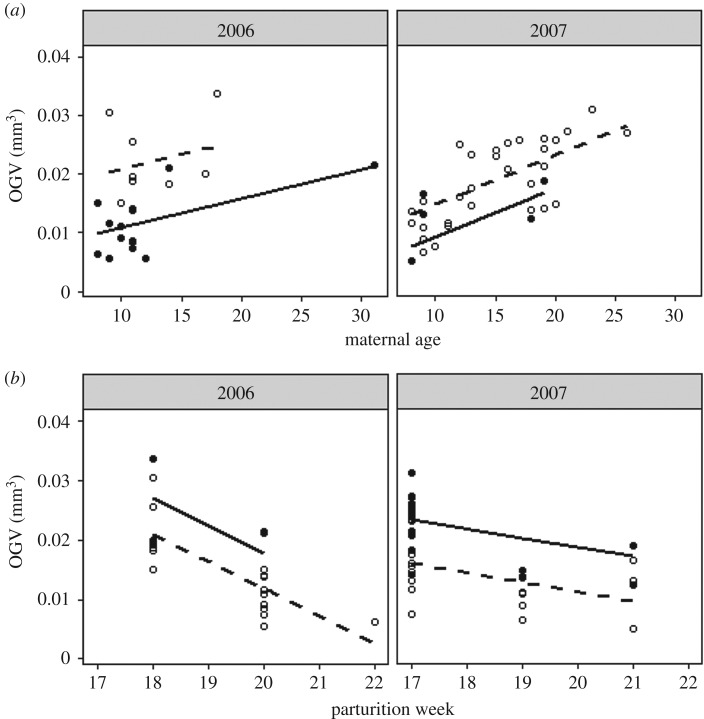
Model prediction lines depicting the relationship between maternal age and OGV for both early (dashed line, weeks 17–19) and late (solid line, weeks 20–22) parturiating females, and the relationship between week of parturition and OGV for both ‘young’ (dashed line) and ‘old’ (solid line, 15+ years) females.

In both years, larvae sampled early in the parturition season had greater OGVs relative to larvae sampled later in the parturition season for a given maternal age (figure 5*a*), but older females consistently provisioned larvae with larger OGVs relative to the larvae of younger females (figure 5*b*) throughout the sampling period. For example, the model defined by equation (3.1) predicted a 56% and an 88% increase in OGV for years 2006 and 2007, respectively, between 10- and 30-year-old females releasing larvae early (week 17) in the parturition season. Late in the parturition season (week 22), the predicted larval OGV for 10- and 30-year-old mothers decreased by 30 to 90% relative to same-age females releasing larvae early in the season; however, the predicted percent increase in globule volume between a 10- and 30-year-old female climbs to greater than 100% in week 22 for both years.

## 4. Discussion

Our analyses show both an effect of maternal age and an effect of release timing on offspring provisioning, with an increase in provisioning between the larvae of young and old females that is maintained despite a decrease in provisioning across all maternal ages through the parturition season. Our findings also suggest a protracted parturition season for the population of older females relative to the population of younger females. Together these results suggest there are multiple determinants of larval provisioning in POP, and that the eventual resolution to the maternal effects paradox will probably require consideration of both maternal age effects and context-dependent maternal effects. Due to the inferential constraints imposed by the observational nature of our field-based study, however, our results do not fully resolve the paradox of maternal age effects in a long-lived marine teleost. Resolution will require experimental research using controlled environmental conditions and longitudinal analyses, as has been done in other taxa [42]. However, the logistics of such a study for a long-lived rockfish are prohibitive.

The finding that larval provisioning decreases through the parturition season could be classified as a context-dependent maternal effect if female POP are responding to environmental changes [43]. Environmental conditions experienced by POP in the northern Gulf of Alaska are characterized by high inter- and intra-annual variation, with the spring transition (typified by an increase in phytoplankton production) beginning across the shelf in late April or May [44]. Increased larval provisioning early in the parturition season could be a fitness advantage if the spring transition begins weeks after the initial larval release date. For evolutionary processes to favour this hypothesized context-dependent maternal effect, the environment of the Gulf of Alaska must vary predictably on the time and spatial scale relevant to larval development, release and dispersal such that the maternal environment during egg formation and development has some correspondence to the larval environment [45]. Our work, however, did not quantify the food resources available during the parturition season, nor did our study evaluate whether or not the maternal environment is correlated to the offspring environment. Additionally, because rockfish are matrotrophic and transfer energy directly to the developing embryo, the degree of larval provisioning may be affected by conditions during larval development prior to parturition in a manner analogous to fecundity downregulation [46] that reflects energy reserves during development. Regulation of offspring provisioning during development would suggest that females with late parturition and low OGV would also have poor body condition. The timing of OGV formation and the degree to which it is regulated, and the relation to environmental conditions experienced by the females, are important topics for future research.

Existing studies investigating the role of maternal effects in population dynamic modelling have generally focused on the relationship between female age and larval provisioning. When hypothesized maternal age effects for two Alaska stocks of POP were considered, after accounting for uncertainties in the relationship between maternal age and reproductive potential, and reproductive potential and recruitment, Spencer *et al*. [47] found decreases in the *per capita* reproductive output that correspond to peak population productivity. In black rockfish (*Sebastes melanops*), large errors in the estimate of lifetime reproductive potential can result if the relationship between larval survival and maternal age is not properly characterized [48]. Murawski *et al*. [49] showed harvest rates based on spawning biomass, without regard for population age structure, overestimated the resiliency of the Georges Bank Atlantic cod (*Gadus morhua*). Simulation analyses using Pacific ocean perch and Pacific cod (*Gadus macrocephalus*) life-history patterns indicate that population productivity is generally overestimated when maternal age effects are not included in population dynamics models, with the degree of overestimation dependent on the magnitude of the maternal effect [50]. Additionally, maternal age can affect rockfish weight-specific relative fecundity [51], which can affect estimates of stock productivity and optimal fishing rates [52]. Future research that seeks to characterize the population-level implications of maternal effects in marine teleosts may be improved by considering both maternal age effects and any potential environmentally driven, context-dependent maternal effects on offspring provisioning, as well as the link between provisioning and offspring quality.

Maternal effects, intrinsic effects of female age together with extrinsic context-dependent effects, could be considered to constitute a portfolio [53] of reproductive strategies, potentially hedging against variable oceanographic conditions and patchily distributed food sources. The loss of maternal age effects via juvenescence of the age structure may increase the susceptibility of a long-lived species to anthropogenic impacts and environmental fluctuations [54]. A meta-analysis of 25 long-lived species in the north temperate and Arctic latitudes found that exploited populations with an extended age structure had higher reproductive rates than age-truncated populations, independent of absolute spawning biomass [55]. A diversified portfolio of reproductive strategies could result in higher reproductive rates, conferring population stability and persistence by buffering against the destabilizing forces of environmental variability and fishing pressure [56,57]. Parsing the portfolio of reproductive strategies to better understand the role of maternal age effects and context-dependent maternal effects in offspring fitness is a necessity for understanding the ultimate impacts on population-level dynamics.

## 5. Conclusion

Our investigation documents the presence of a maternal age effect on release timing and an effect of maternal age and release timing on larval provisioning in a long-lived teleost. We have shown that when one element of environmental context is considered (timing of parturition), a positive relationship between maternal age and offspring provisioning is maintained. However, we were unable to resolve the paradox of maternal age effects as presented by Marshall *et al*. [9]. Field sampling allowed us to evaluate maternal effects within the environmental context of breeding females, and therefore investigate an argument for the environmental context of maternal effects using offspring release timing as a proxy for changing environmental conditions. Quantifying the environmental conditions throughout the entire breeding cycle of the female, as well as the release environment of the offspring, will further clarify the role of both maternal age effects and context-dependent maternal effects. The evolutionary paradox of the maternal age effect does not uniformly dismiss the existence nor importance of maternal age effects, but prompts a closer examination of reproductive strategies that will ultimately support the research of conservation biologists and fisheries scientists tasked with maintaining healthy populations of long-lived marine teleosts in an increasingly variable environment.

## Data accessibility

The datasets supporting this article are available in ScholarsArchive@OSU, hosted by Oregon State University, Corvallis, OR, USA. (doi:10.7267/N9KS6PHM).
